# Optimal Design of High-Strength Ti‒Al‒V‒Zr Alloys through a Combinatorial Approach

**DOI:** 10.3390/ma11091603

**Published:** 2018-09-04

**Authors:** Di Wu, Yueyan Tian, Ligang Zhang, Zhenyu Wang, Jinwen Sheng, Wanlin Wang, Kechao Zhou, Libin Liu

**Affiliations:** 1 School of Material Science and Engineering, Central South University, Changsha 410083, China; wudi080133@163.com (D.W.); yueyantian@csu.edu.cn (Y.T.); ligangzhang@csu.edu.cn (L.Z.); zhenyuwang@csu.edu.cn (Z.W.); shengjinwen@csu.edu.cn (J.S.); 2 Key Laboratory of Non-Ferrous Metallic Materials Science and Engineering, Ministry of Education, Changsha 410083, China; 3 School of Metallurgy and Environment, Central South University, Changsha 410083, China; wanlin.wang@csu.edu.cn; 4 State Key Laboratory of Powder Metallurgy, Changsha 410083, China

**Keywords:** combinatorial alloy design, diffusion multiple, Ti6Al4VxZr, microstructure and mechanical properties, phase transformation

## Abstract

The influence of various Zr contents (0–45 wt.%) on the microstructure and mechanical properties of Ti6Al4V alloy was investigated through a combinatorial approach. The diffusion multiples of Ti6Al4V–Ti6Al4V20Fe–Ti6Al4V20Cr–Ti6Al4V20Mo–Ti6Al4V45Zr were manufactured and diffusion-annealed to obtain a large composition space. Scanning electron microscopy, electron probe micro-analysis, and a microhardness system were combined to determine the relationships among the composition, microstructure, and hardness of these alloys. The Ti–6Al–4V–30Zr alloy was found to contain the thinnest α lath and showed peak hardness. X-ray diffraction and transmission electron microscope results indicated that after quenching from the β-field, the metastable α″-phase formed; moreover, at the secondary aging stage, the metastable α″-phase acted as precursor nucleation sites for the stable α-phase. The bulk Ti6Al4V30Zr alloy was manufactured. After aging at 550 °C, the alloy showed excellent balance of strength and ductility, and the tensile strength was 1464 MPa with a moderate elongation (8.3%). As the aging temperature increased, the tensile strength and yield strength of the alloys rose, but the total elongation decreased. The lamella thickness and volume fraction of the α-phase were the major factors that had great impacts on the mechanical properties.

## 1. Introduction

Titanium and its alloys are widely used in the aerospace industry due to their exceptional strength-to-weight ratio, high crack propagation resistance, outstanding fatigue properties, considerable hardening ability, and good corrosion resistance [1,2]. Ti–6Al–4V is one of the most important and widely used titanium alloys in the aerospace industry because of its optimum comprehensive mechanical properties. However, its application is limited because of its low strength [3,4,5]. These problems can be overcome by adding alloying element(s) to the Ti–6Al–4V alloy to enhance its strength. Hence, new alloy systems need to be designed to satisfy the requirements of the new environment.

Zirconium (Zr) and titanium (Ti) possess similar physicochemical properties and belong to the same main group in the periodic table of chemical elements. Zirconium and Ti can form complete solid solution for hcp-structure low-temperature α-phase and bcc-structure high-temperature β-phase [6]. Previous research has shown the apparent influences of alloy element Zr on the microstructure and mechanical properties of Ti alloys. Schmuki [7], Liu [8], and Ho [9] have investigated the microstructure and mechanical properties of Ti–Zr binary alloys, and the results have shown that the phase composition and mechanical properties of these binary alloys varied remarkably with changes in Zr content. Moreover, previous studies [10,11,12,13] showed that Zr had significant influence on the properties of many multicomponent Ti alloys. The influence of Zr content on the structure and properties of ternary Ti–Nb–Zr alloys was investigated by Zhou [10], and the result showed that increasing Zr content decreased the strength of the alloy but increased the ductility. Chen [11] investigated the effect of Zr on the structure and mechanical and thermal properties of Ti–Al–N-based coating, and noted that the addition of Zr promoted the formation of cubic domains and retarded the formation of stable wurtzite AlN, which led to high hardness during thermal annealing. Liang [12] investigated the microstructure and mechanical properties of Ti–5Al–3V–30Zr alloy that has undergone various annealing heat treatments, and the alloy has shown good mechanical properties. However, the strengthening mechanism and phase transformation of the Ti–5Al–3V–30Zr alloy has not yet been reported. Recently, a series of Ti–6Al–4V–xZr (0 ≤ x ≤ 20) alloys were investigated by Jing [13], and Zr was selected as a partial substitutable element for Ti in Ti–6Al–4V alloys. The new Ti–6Al–4V–20Zr alloy exhibited good compositive performances (i.e., balance of strength and ductility), showing its great potential for high-strength structural applications in the aerospace industry. Zirconium could be a suitable element to modify Ti and Ti alloys. 

Only partial alloy compositions of the Ti–6Al–4V–xZr system that may have a relatively good performance have been investigated. Thus, the study of the Ti–6Al–4V–xZr system with a wide range of composition provides many opportunities for the optimization of alloy composition. The conventional one-alloy-at-a-time methods are time-consuming and costly, and may prove hard to enforce. Therefore, a combinatorial approach was used in our research.

The diffusion-multiple approach has been used to determine very complex phase diagrams for many systems [14,15]. In addition to phase diagram mapping, the diffusion multiples have the ability to create continuous composition gradients of solid solutions, which could be used to map material properties with different kinds of micro-probes [16,17]. Through this approach, Zhang et al. [18] have established a database of composition-dependent elastic modulus and hardness of the Ti–Zr–Ta systems and provided an essential guide for the alloy design of new-type bio-titanium alloys. Chen [19] mapped the microstructural and mechanical data of Ti–Al–Mo alloys. Wu [20] also investigated the effects of Cr addition on the microstructures and hardness of Ti6Al4V alloy, and the result can be used to accelerate the discovery of high-strength titanium alloys.

The purpose of this work was to investigate the effects of Zr on the microstructures and mechanical properties of Ti–6Al–4V alloy in an effort to develop a new high-strength titanium alloy for aerospace application. Firstly, the alloying element Zr was added to increase the strength of the Ti–6Al–4V alloy, and then the plasticity of the alloy could be improved by thermal processing and heat treatment. The microstructure, mechanical properties, and phase transition of Ti–6Al–4V–30Zr alloy that has undergone various annealing heat treatments were also investigated.

## 2. Materials and Methods

**Figure 1:**
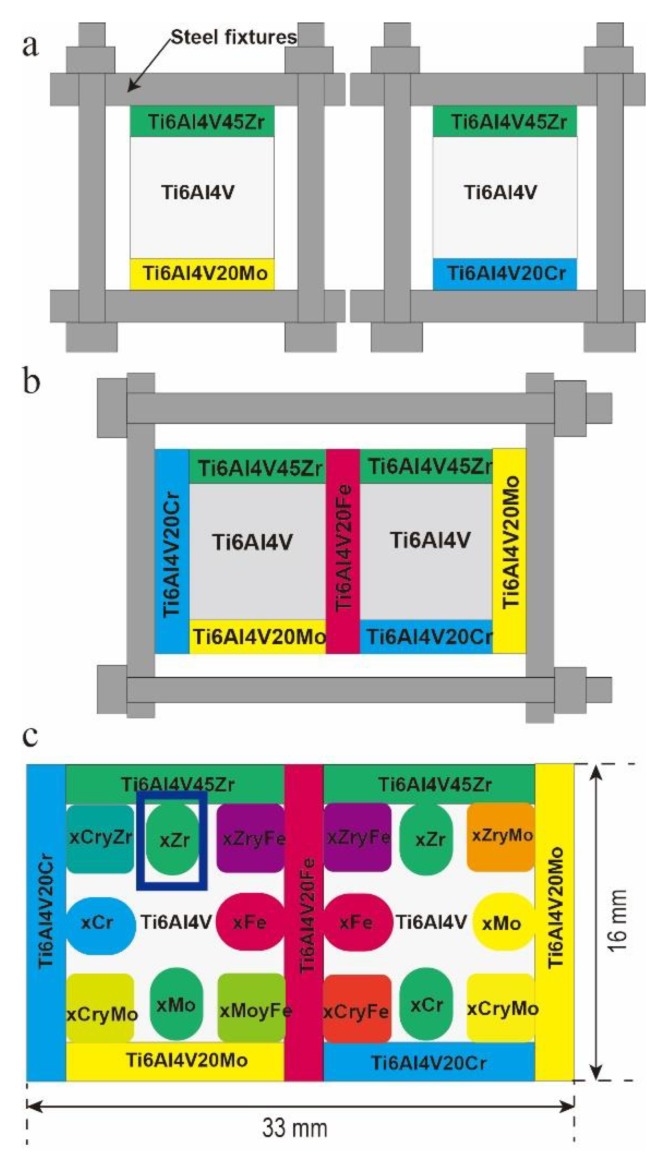
The fabrication process of Ti6Al4V-Ti6Al4V20Fe-Ti6Al4V20Cr-Ti6Al4V20Mo-Ti6Al4V45Zr diffusion multiple: (**a**) two diffusion multiples with three bricks of alloys were manufactured with steel fixtures; (**b**) Ti6Al4V-Ti6Al4V20Fe-Ti6Al4V20Cr-Ti6Al4V20Mo-Ti6Al4V45Zr diffusion multiple manufactured with steel fixtures; and (**c**) cross-sectional schematics of the Ti6Al4V-Ti6Al4V20Fe-Ti6Al4V20Cr-Ti6Al4V20Mo-Ti6Al4V45Zr diffusion multiples.

A diffusion multiple is a combination of several diffusion couples and triples arranged in one sample according to a designed geometry [21,22,23]. The Ti6Al4V–Ti6Al4V20Fe–Ti6Al4V20Cr–Ti6Al4V20Mo–Ti6Al4V45Zr diffusion multiple was manufactured using the method shown in Figure 1. The continuous and large-range composition gradients of the alloying elements were formed by long-time diffusion annealing. Combined with different kinds of microprobes, we rapidly analyzed the different properties in the large composition space to build the composition-microstructure-property relationship. In this study, the diffusion-multiple approach was employed to investigate the influence of using varied Zr contents on the structure and mechanical properties of Ti–6Al–4V alloy.

All parts for the diffusion multiples were cut via electrical discharge machining (EDM, Central South University, Changsha, China). The dimensions of the pieces are shown in Figure 1a. The diffusion multiple included two Ti‒6Al‒4V bricks with dimensions of 12 mm × 12 mm × 10 mm; two Ti‒6Al‒4V‒45Zr, one Ti‒6Al‒4V‒20Cr, and one Ti‒6Al‒4V‒20Mo plates with dimensions of 12 mm × 12 mm × 3 mm; and one Ti‒6Al‒4V‒20Cr, one Ti‒6Al‒4V‒20Fe, and one Ti‒6Al‒4V‒20Mo plates with dimensions of 16 mm × 12 mm × 3 mm. The materials used for the diffusion-multiple included Ti‒6Al‒4V and Ti‒6Al‒4V‒45Zr. The materials were prepared from high purity Ti (99.95%), Zr (99.7%), Al (99.99%), and V (99.95%) using arc-melting technique in an argon atmosphere. The actual compositions of Ti‒6Al‒4V and Ti‒6Al‒4V‒45Zr, which were measured via inductively coupled plasma atomic emission spectrometry (Northwest Institute for Non-ferrous Metal Research, Xi’an, China), are listed in Table 1.

**Figure 2:**
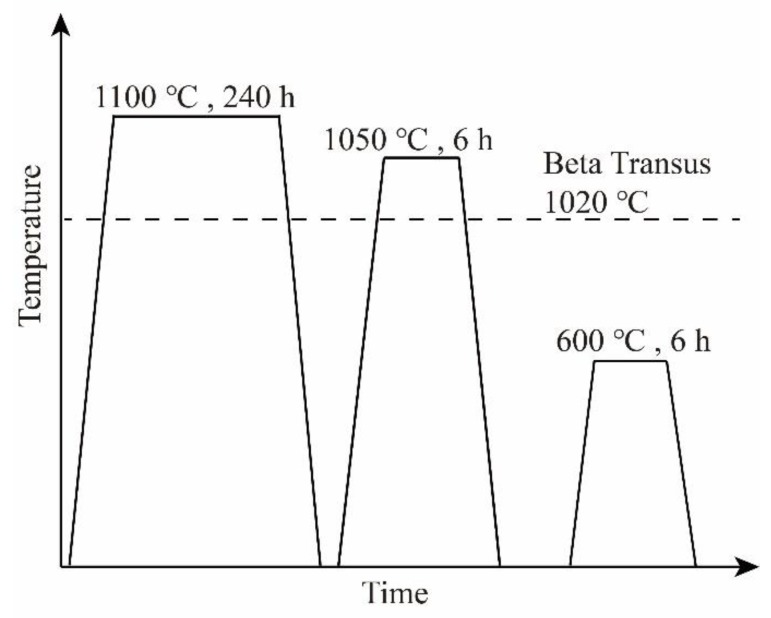
Heat treatment schedule for the Ti6Al4V‒Ti6Al4V20Fe‒Ti6Al4V20Cr‒Ti6Al4V20Mo‒Ti6Al4V45Zr diffusion multiple (this figure is not to scale.).

The bonding faces of the diffusion multiple were mechanically polished via a standard metallographic procedure to a final level of 0.3 µm alumina powder. As shown in Figure 1a, two diffusion multiples Ti6Al4V–Ti6Al4V20Mo–Ti6Al4V45Zr and Ti6Al4V–Ti6Al4V20Cr–Ti6Al4V45Zr with three bricks of alloys were clamped in steel fixtures and diffusion-annealed at 1000 °C for 4 h in a vacuum (about 10^−3^ Pa). Afterward, the bonding faces of the diffusion multiple with three bricks were ground and polished, and the diffusion multiple with three bricks of alloys and two bricks of diffusion multiple were clamped in steel fixtures again, as shown in Figure 1b. Afterward, the diffusion multiples were sealed into an evacuated quartz tube and annealed at 1100 °C for 240 h to obtain a wide range of compositions. In this work, we will investigate the effects of Zr content on the microstructure and properties of Ti–6Al–4V alloy, which is located in the blue box area in Figure 1c. Then, the samples were solution-treated at the β-phase regions (1050 °C) for 6 h and water quenched. The quenched samples were aged at 600 °C and 650 °C for 6 h following water quenching. The heat treatment system of the diffusion multiples is shown in Figure 2. Finally, the diffusion multiples were mounted, ground, and polished by standard metallographic techniques.

Before microhardness tests, the binary and ternary diffusion region range was determined with electron probe micro-analysis (EPMA Central South University, Changsha, China) on a JEOL JXA-8230 microprobe with 20 kV, 20 nA and 40° take-off angle. Microhardness system was used to test hardness (BUEHLER5104 Central South University, Changsha, China), the intervals of the indentations were 100 μm, and the applied load was 500 mN with a hold time of 15 s. Field emission scanning electron microscope (FESEM Central South University, Changsha, China) was used to characterize the microstructure of the diffusion multiples (JEOL-JSM 7001F Central South University, Changsha, China). Finally, the composition on both sides of the indentation was measured by quantitative EPMA analysis again. The lamellar thickness of the α-phase was obtained by calculating the average values of those incising the diagonals of the SEM micrographs (ASTM: E112-12 Central South University, Changsha, China).

The alloy buttons of Ti‒6Al‒4V‒30Zr were prepared from high purity Ti (99.95%), Zr (99.7%), Al (99.99%), and V (99.95%) via arc-melting technique in an argon atmosphere. The buttons (~10 g each) were heat-treated the same as the diffusion multiple samples. Phase structures were examined through X-ray diffraction (XRD) (XRD-6000, Shimadzu, Japan). Transmission Electron Microscopy (TEM Central South University, Changsha, China) was used to investigate phase transformation and phase composition (Titan G260-300 Central South University, Changsha, China).

The bulk alloy of Ti‒6Al‒4V‒30Zr was prepared using sponge titanium, pure Zr, pure Al, and Ti–V master alloys through a double-vacuum arc-melting process. The ingot with 200 mm in length and 160 mm in diameter was first β-fogged down at 1000 °C and subsequently forged at 820 °C to 40 mm thick and 80 mm wide bars. The β-transus temperature of the alloy was determined metallographically and was found to be 800 ± 5 °C. The samples from the as-forged material were first solution-treated at 820 °C for 0.5 h and followed by air cooling. Afterward, the samples were aged at 500 °C, 550 °C, 600 °C, and 650 °C for 6 h and followed by air cooling. Optical microscopy (OM Central South University, Changsha, China) was used to characterize the forged alloy, which was etched in Kroll’s reagent (3% HF + 9% HNO_3_ + 88% H_2_O). The microstructures were investigated using FESEM (JEOL-JSM 7001F Central South University, Changsha, China). Tensile testing was carried out on a MTS810 testing machine (Central South University, Changsha, China) and driven at a crosshead speed of 2 mm/min. Bone-shaped plate specimens with a 25 mm gauge length, 4 mm gauge width, and 3 mm gauge thickness were prepared for tensile tests.

## 3. Results and Discussion

### 3.1. Microstructure of Ti6Al4VxZr Alloys Taken from Ti6Al4V–Ti6Al4V45Zr Diffusion Multiple

**Figure 3:**
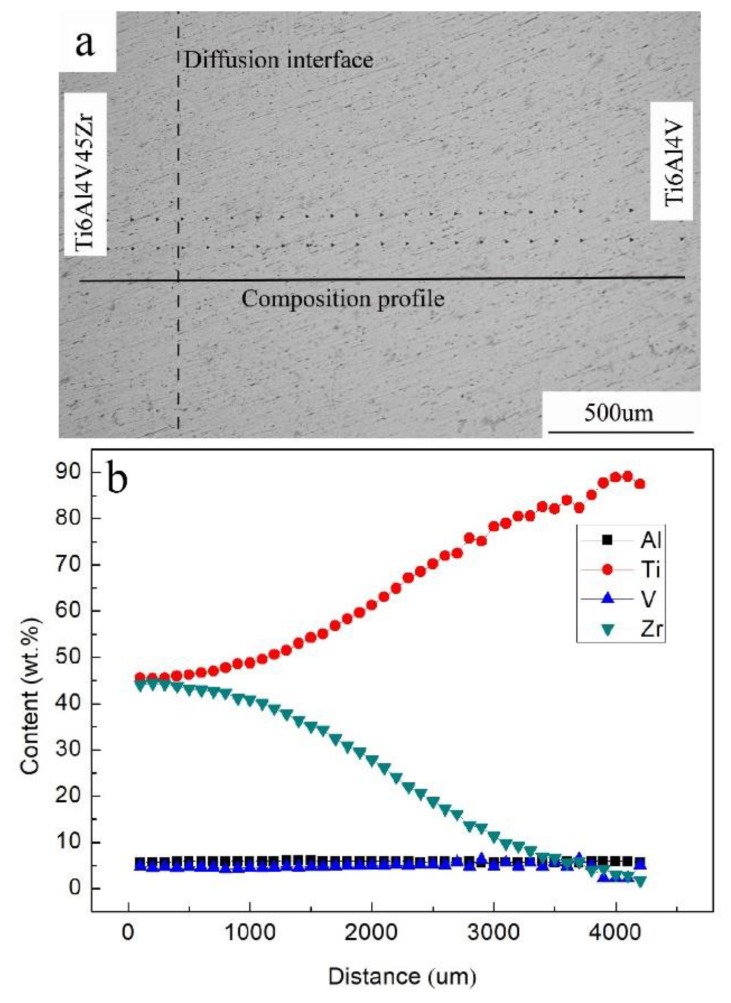
(**a**) Optical image of hardness indentation grid at the Ti6Al4V–Ti6Al4V45Zr diffusion multiple; (**b**) variation in the composition as a function of distance for the Ti6Al4V–Ti6Al4V45Zr diffusion multiple.

Figure 3 plots the composition changes as a function of diffusion distance for the Ti6Al4V–Ti6Al4V45Zr system. Zirconium content reduced gradually to 0 with the increase in diffusion distance. The Zr atom mainly substituted Ti, and the contents of Al and V almost remained constant.

**Figure 4:**
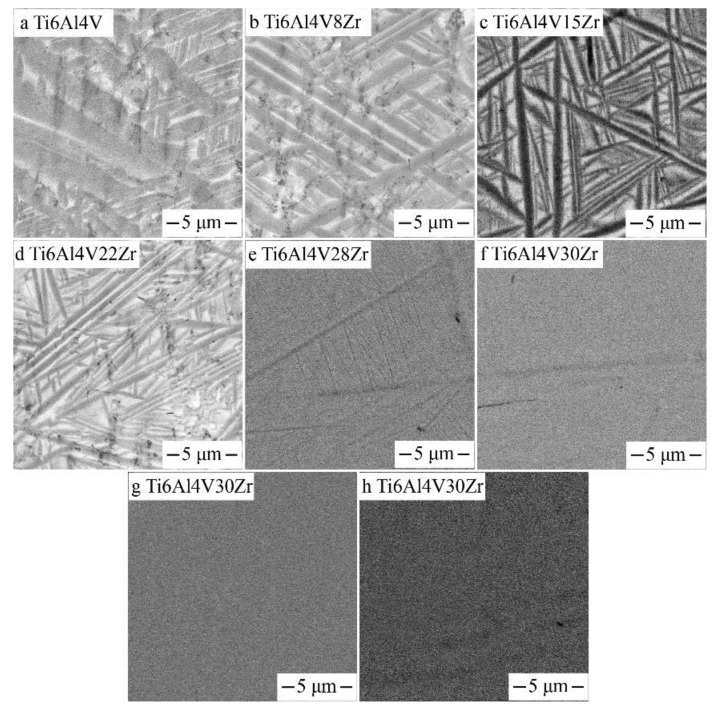
SEM, backscattered electron images of beta annealed Ti6Al4VxZr alloys taken from the Ti6Al4V–Ti6Al4V45Zr diffusion multiple.

Figure 4 shows the backscatter electron micrographs of the Ti‒6Al‒4V‒xZr alloys. These SEM micrographs were taken from different places of the diffusion multiples. The Zr content of the Ti‒6Al‒4V‒xZr alloys were around 8, 15, 22, 28, and 30 wt.%. As shown in Figure 4a,b, the Ti‒6Al‒4V and Ti‒6Al‒4V‒8Zr displayed coarse α′ martensitic lath. When the Zr content increased to 15–22 wt.%, the structure morphology of the alloys was similar to the Ti‒6Al‒4V alloy, and the thickness and fractional volume of the martensitic lath decreased. When 28 wt.% Zr was added, very fine martensitic laths were observed (Figure 4e). When the alloy contained 30 wt.% or more Zr, no α′ martensitic lath was visible in the SEM images, and β-phase became the dominant phase (Figure 4f). The β-phase was entirely retained during quenching when the Zr content exceeded 30 wt.%, perhaps because the alloying element Zr acted as a weak β stabilizer, which will suppress the formation of α′ martensite. The alloys contained 38 and 45 wt.% Zr also exhibit a single β phase, as shown in Figure 4g,f. This result indicated that the martensitic start (Ms) temperature dropped below room temperature after 30 wt.% Zr was added. Importantly, ω and α″ phases are too fine to be observed through the backscattered image. Previous research has shown that the martensite phase transformation from β→α″ takes place during water quenching in Ti‒Al‒V‒Zr alloys [12]. This result means that the quenched Ti‒6Al‒4V‒30Zr alloy was comprised mainly of the β-and α″ phases, which could be verified in the TEM results.

**Figure 5:**
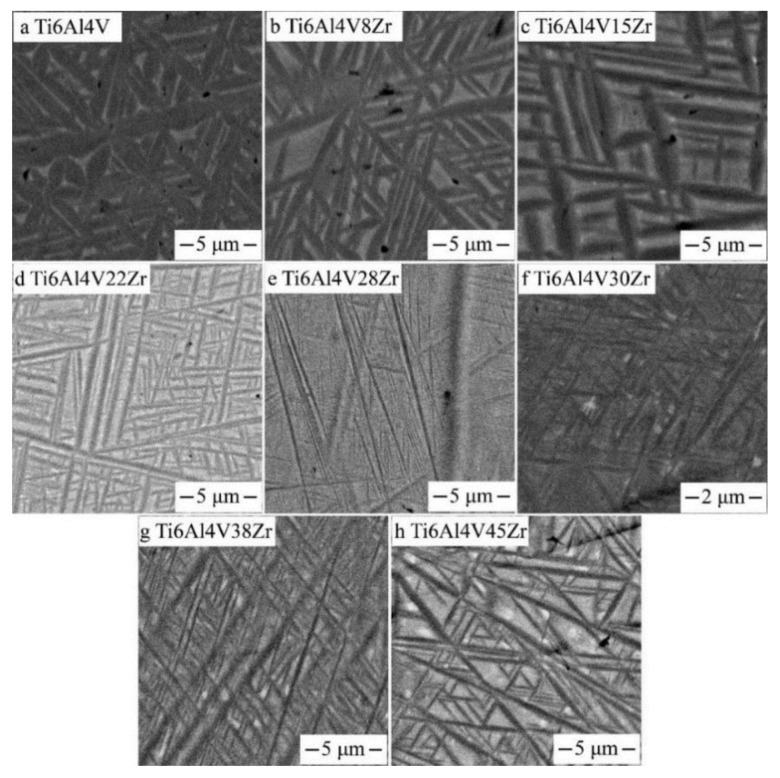
SEM, backscattered electron images of Ti6Al4VxZr alloys after aging at 600 °C taken from the Ti6Al4V–Ti6Al4V45Zr diffusion multiple.

The influences of various Zr contents on the microstructures of the Ti‒6Al‒4V alloy after aging at 600 °C are shown in Figure 5. These SEM images were taken from the diffusion multiple and the contents of the Zr from 0–45 wt.%. As shown in Figure 5, the volume fraction of the α-phase decreased monotonously, and the lamellar thickness of the α-phase initially decreased and then increased as Zr contents increased. As shown in Figure 5a, the Ti‒6Al‒4V alloy displays the coarse basket-weave microstructure, and the lamellar thickness of the α-phase was approximately 1000 nm. Based on the increase of Zr contents, the lamellar thickness of the α-phase remarkably decreased. When the alloy contained 30 wt.% Zr, the alloy displayed ultrafine α grains compared with other alloys. The nanoprecipitation phase were hardly observable through SEM microscopy. However, further increasing the Zr content increased the lamellar thickness of the α-phase.

**Figure 6:**
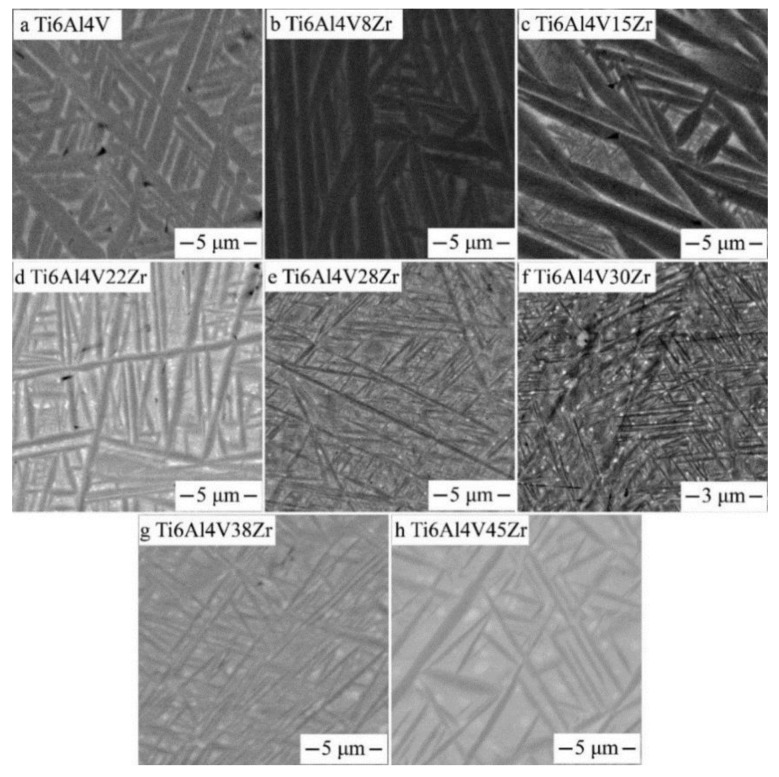
SEM, backscattered electron images of Ti6Al4VxZr alloys after aging at 650 °C taken from the Ti6Al4V–Ti6Al4V45Zr diffusion multiple.

The series of SEM micrographs showing the α precipitate morphologies for various Zr contents after aging at 650 °C for 6 h is shown in Figure 6. At 650 °C, the precipitates were coarser and had less volume fraction than in the lower aging temperatures. Moreover, the finest acicular α precipitates appeared in the alloy with compositions of approximately 30 wt.% Zr.

**Figure 7:**
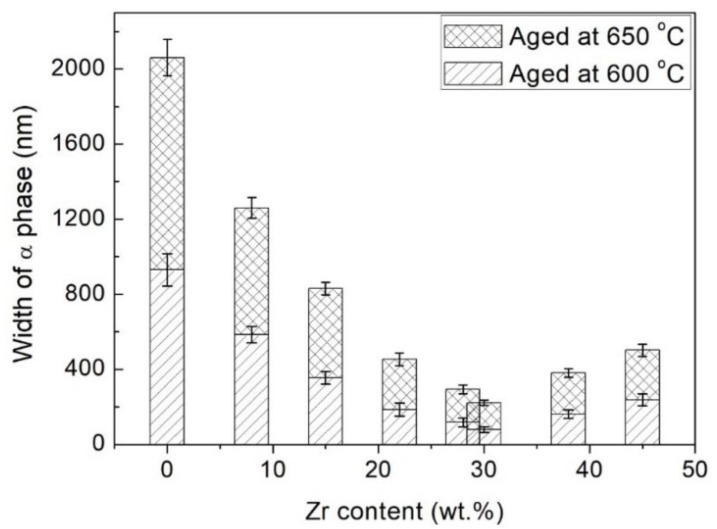
Width of α phase as function of Zr content after aging at 600 °C and 650 °C.

The quantitative statistics of the lamellar thickness of the α-phase based on digital image processing are shown in Figure 7. After aging at 600 °C and 650 °C, the α lamellar thickness of Ti6Al4V reached to 1000 and 1200 nm, respectively. Increasing the Zr content to 30 wt.% decreased the thickness of the α-phase to 50 and 70 nm, respectively, and the Ti‒6Al‒4V‒30Zr alloy had ultrafine grains. When the Zr content was below 30 wt.%, the martensite transition temperature was below room temperature. After quenching from the β-field, an α′ martensite distribution was obtained. The microstructures of the aged alloys were remarkably dependent on the martensite quenching structure. During aging at 600 °C for 6 h, Ti‒6Al‒4V‒xZr alloys underwent phase transition between α′ martensite to α-phase, and the mutual diffusion of Al, V, and Zr elements occurred between α- and β-phases. When Zr content increased to 30 wt.%, and the metastable α″- or ω-phases occurred in the β-matrix after quenching. These nano-sized particles may act as heterogeneous nucleation sites for α-phase and increase the nucleation rate of the α-phase during the aging treatment. The ultrafine structures of Ti‒6Al‒4V‒30Zr alloy resulted from the increase of α-nucleation rate. When the Zr content was increased to 38 wt.% or greater, only the retained β-phase was observed, and no other structures units acted as heterogeneous nucleation site for α-phase, which resulted in the decrease of α-nucleation rate and coarser α-phase.

### 3.2. Influence of the Zr on the Hardness after Beta-Annealing and after Aging

**Figure 8:**
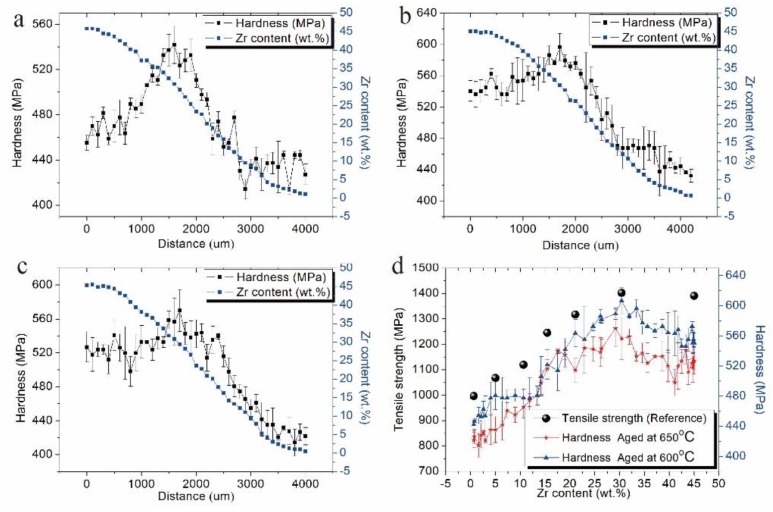
Composition-hardness relationship of the series Ti6Al4VxZr alloys: (**a**) after beta annealing; (**b**,**c**) after aging at 600/650 °C; (**d**) hardness of aged alloys compared with reference.

Figure 8 shows that the hardness and composition of quenched and aged Ti‒6Al‒4V‒xZr alloys change as a function of diffusion distance. The results show that Zr had notable influence on hardness. The hardness of quenched alloys increased with Zr content and peaked as the Zr content in Ti‒6Al‒4V reached approximately 30 wt.%. When the content of Zr was 30 wt.%, its hardness was 541 MPa, which was approximately 30% higher than that of the Ti‒6Al‒4V alloy. This result indicates that the hardness of quenched alloys strongly depends on their microstructures. As mentioned above, the quenched alloy exhibited the nanoscale and microscale martensitic lath when the Zr content was approximately 30 wt.%. The fine martensitic lath generated a mass of α″/β-interfaces, which hindered dislocation motion and improved alloy hardness [24]. As described above, Zhang et al. [25] found the martensite α″ and the high strength in the Ti‒5Al‒3V‒30Zr alloy and reported that the metastable α″ martensite can increase the strain-hardening exponent, resulting to the increase of hardness. When the alloys contained 45 wt.% Zr, the mount of α″ precipitates decreased or even disappeared, which may result to decrease in hardness. This finding agreed with the findings in Majumdar′s [26] study, where the hardness dropped to a minimum when the Zr content was about 50 wt.% in the beta-annealed titanium alloys.

After aging at 600 °C, the hardness increased from 439 MPa to 605 MPa as Zr content increased from 0 wt.% to 30 wt.% and then decreased, as shown in Figure 8b. After aging at 650 °C, the hardness decreased slightly. This result was mainly due to the increase in α-lath thickness and decrease in the volume fraction of the α-phase, (i.e., hardness tends to change after aging at 650 °C similar to that after aging at 600 °C). The Ti‒6Al‒4V‒30Zr alloy displayed a fine scale dispersion of the α-phase and showed a hardness of about 605 MPa, which was a 35% improvement upon that of the Ti‒6Al‒4V alloy. The strengthening mechanisms were chiefly precipitation, grain refinement, and solid solution reinforcement. Compared with the Ti‒6Al‒4V alloy, the Ti‒6Al‒4V‒30Zr alloy had a larger number of α precipitates after the addition of 30 wt.% Zr, and the α lath was finer and generated a mass of α/β-interfaces and close interlaminar spacings, which effectively hindered dislocation-slipping according to the Orowan relationship. Previous research [27,28,29,30,31,32] has shown that the α/β-phase boundary in titanium alloys fall into two obvious categories, namely, coherent and incoherent phase boundaries. In titanium alloys, the α/β-phase boundary with fine and uniform α-phase is the coherent phase boundary, and the phase boundary with coarse microstructures belongs to the incoherent phase boundary. The interfacial bonding strength of the coherent phase boundary is stronger than that of the incoherent phase boundary, which gives the hardness and finest α lamellar qualities of the Ti‒6Al‒4V‒30Zr alloy compared with other alloys. The chemical compositions of the α- and β-phases after aging at 600 °C were also determined. Table 1 shows that the α- and β-phases have almost the same Zr contents, which were homogeneously distributed inside the α- and β-phases. Previous studies have shown that Zr mainly functioned as a solution-strengthening element. High concentration of Zr solute atoms in the α- and β-phase results in distorted lattice and increased dislocation density due to the different atomic dimensions between Zr and Ti. The strength enhancement of the Ti‒6Al‒4V‒30Zr alloy could also be caused by solid solution-strengthening [33,34].

The tensile strengths of a series of Ti‒Al‒V‒Zr alloys are shown in Figure 8d. The composition of the alloys were Ti‒6Al‒4V, Ti‒6Al‒4V‒5Zr, Ti‒6Al‒4V‒10Zr, Ti‒6Al‒4V‒15Zr, Ti‒6Al‒4V‒20Zr [13], Ti‒5Al‒3V‒30Zr [12], and Ti‒5Al‒3V‒47Zr [35]. The tendency of the diffusion multiple to change in hardness was comparable with the strength variable trend obtained using the alloy method. Zhang et al. [12] reported that Ti5Al3V30Zr had the largest tensile strength of 1407 MPa among the Ti‒Al‒V‒Zr alloys. As described above, Ti‒6Al‒4V‒30Zr is likely to be the most promising high-strength candidate alloy among the Ti‒6Al‒4V‒xZr alloys.

### 3.3. XRD and STEM Results of Ti‒6Al‒4V‒30Zr Alloy

**Figure 9:**
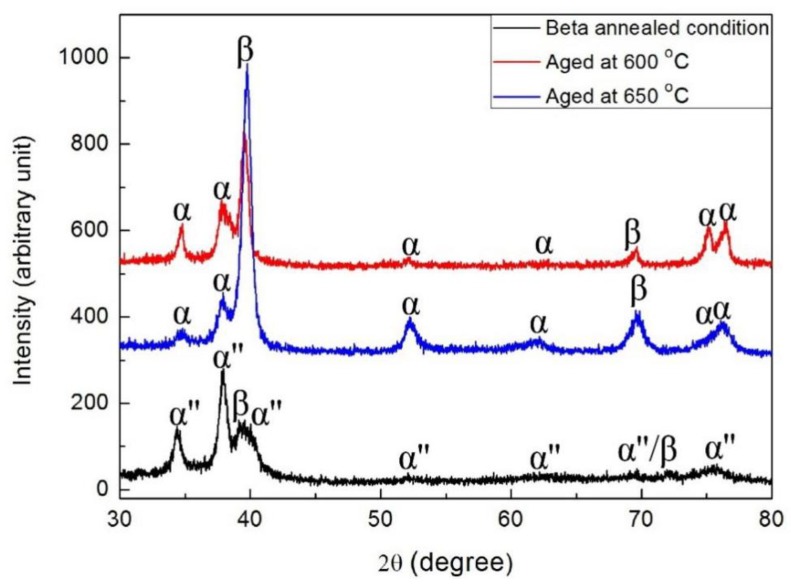
XRD patterns of solution-treated and as-aged Ti6Al4V30Zr alloy.

Figure 9 shows the XRD spectrums of the as-quenched and as-aged Ti‒6Al‒4V‒30Zr alloys. The results show that the as-quenched microstructures of Ti‒6Al‒4V‒30Zr were α″ lath martensite and β-matrix. No other phase was observed at this stage. The hexagonal close-packed structure of the α-phase was seen after aging at 600 °C and 650 °C for 6 h. The α-phase should have evolved from the quenched α″ phase.

**Figure 10:**
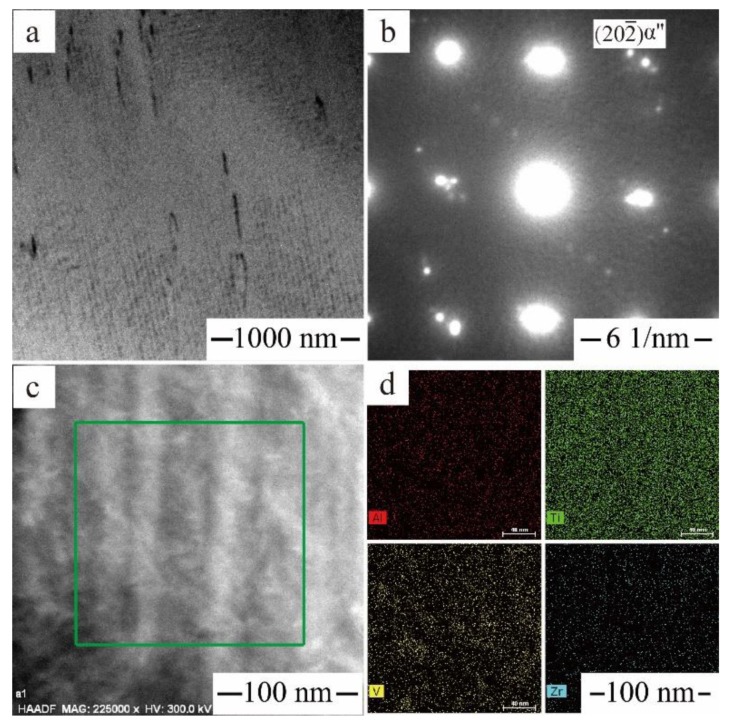
Microstructural feature of the Ti6Al4V30Zr alloy after beta annealing at 1050 °C: (**a**) bright field (BF) image showing α″ laths; (**b**) SAD (selected area electron diffraction) pattern with [113] β zone axis; (**c**) HAADF-STEM images; (**d**) The EDS mapping of Zr, V, Al, and Ti atoms.

Figure 10 shows the microstructural features of Ti‒6Al‒4V‒30Zr alloy in the quenched condition. The nanoscale precipitates of the α″ phase were homogeneously distributed and clearly visible in the bright-field image. These α″ laths had two orientations, which were perpendicular to each other. The presence of α″ nanosize laths was not detected by SEM, which may be due to the absence in compositional variation between the α″- and β-matrix. Figure 10b shows the [011] β-zone axis electron diffraction patterns with additional reflections next to {202} positions, which arose from the α″ phase. In Figure 10b, extra diffraction spots were observed and identified as that of the orthorhombic α″ phase besides diffraction spots of the original β-phase. A high-angle annular dark field (HAADF) image recorded from one of the additional α″ reflections is shown in Figure 10c. This image confirms the presence of nanometer-scale, quenched-in α″ precipitates within the β-grains. The average thickness of α″ laths is around 30 nm. Guo et al. [36] have also reported the metastable α″ phase in 40.2Ti–51.1Zr–4.5Al–4.2V alloy, with the mean width of α″ laths as ~46 nm. Liang et al. [25] have found the single martensite α″ in the Ti‒5Al‒3V‒30Zr alloy and reported that the metastable α″ martensite transformation arose from tensile stress and could increase the strain-hardening exponent significantly, which will result in high hardness. In the aged stage, the α-phase is generated, and the fine α″ martensite may serve as the nucleation site of the α-phase. Figure 10c shows the EDS mapping result from this sample, exhibiting the atoms distribution in the green square. The results indicate that the compositional fluctuations were not obvious in the Zr, Al, and V content, and all the alloy components were evenly distributed in the beta-annealed condition. This result indicates a shear-type β-to-α″ phase transformation without composition difference from the β-phase.

**Figure 11:**
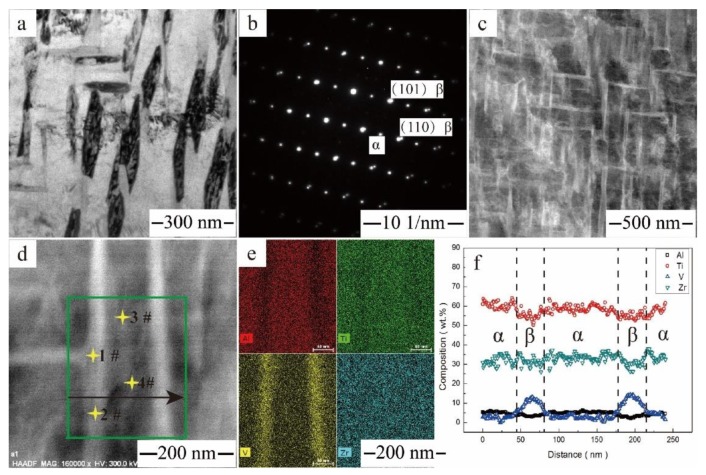
Microstructural feature of the Ti6Al4V30Zr alloy after aging at 600 °C: (**a**) bright field (BF) image; (**b**) SAD pattern with [111] β zone axis; (**c**,**d**) HAADF-STEM images showing α phase; (**e**) the EDS mapping of Zr, V, Al, and Ti atoms; (**f**) the composition profiles along the arrows shown in (**d**).

The microstructural feature of the Ti‒6Al‒4V‒30Zr alloy aged at 600 °C is shown in Figure 11. The nanometer-scale α precipitates were homogeneously distributed within β-grains as clearly shown in the bright-field images in Figure 11a. Compared with the quenched condition, the nanoscale α-phases also had two orientations, which were perpendicular to each other. The same crystal orientation of α and α″ laths indicates that α″ precipitates act as heterogeneous nucleation sites for the α precipitates in Ti‒6Al‒4V‒30Zr. The dimensions for the secondary α-phase were ~200 nm long and ~60 nm wide, and the spacing of the α precipitates in the β-matrix was ~20 nm in width. The nanoscale β-laths was another reason that resulted to the peak hardness of the Ti‒6Al‒4V‒30Zr alloy. Figure 11b shows the [111] β-zone axis electron diffraction patterns with additional reflections at 1/2 {110} β-positions, as well as reciprocal lattice streaking. These additional reflections were arose from secondary α-phases.

**Figure 12:**
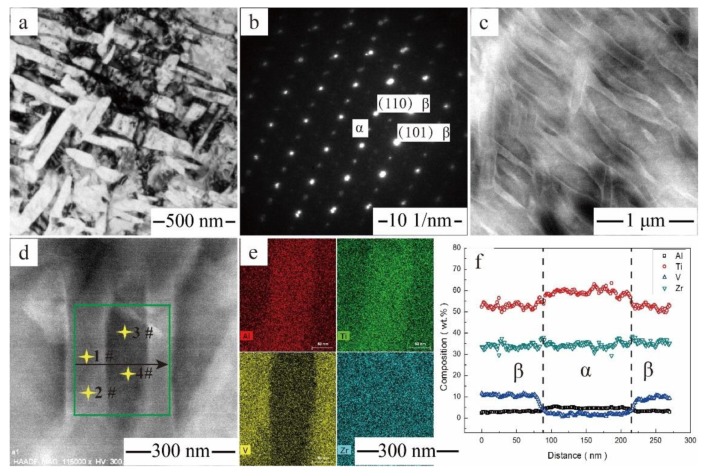
Microstructural feature of the Ti6Al4V30Zr alloy after aging at 650 °C: (**a**) BF image; (**b**) SAD pattern with [111] β zone axis; (**c**,**d**) HAADF-STEM images showing α phase; (**e**) the EDS mapping of Zr, V, Al, and Ti atoms; (**f**) the composition profiles along the arrows shown in (**d**).

Figure 11c shows a HAADF-STEM image of the Ti‒6Al‒4V‒30Zr alloy after aging at 600 °C. Figure 11d,e show EDS atom maps with square regions of the aged sample containing an α plate, which has a distinct color. A compositional profile across the α plate is shown in Figure 11f. A comparison of the chemical composition of the α- and β-phases is given in Table 2. The EDS mapping reveals an enrichment in Al and depletion in V in the α-phase and an enrichment in V and depletion in Al in the β-phase (i.e., Al is an α-stabilizer and V is a β-stabilizer). Zirconium was homogenized in the α- and β-phases (i.e., Zr is a neutral element). The microstructural feature of the Ti‒6Al‒4V‒30Zr alloy aged at 650 °C is shown in Figure 12. Compared with the alloy aged at 600 °C, the precipitates of the Ti‒6Al‒4V‒30Zr were coarser, and the dimensions for the secondary α-phase were ~200 nm long and ~110 nm wide. The chemical compositions of the α- and the β-phases after aging at 650 °C were also determined and listed in Table 2, which shows that the α- and β-phases have almost the same Zr contents, and the Zr was homogeneously distributed inside the α- and β-phases.

As shown in Figure 11 and Figure 12, the aged Ti‒6Al‒4V‒30Zr alloy displays an ultrafine-grained microstructure. The α precipitates were nucleated preferentially at the grain boundaries, such as prior β-grain boundaries and β/ω interfaces, and other lattice defects, such as dislocations and sub-boundaries [37,38]. The composition of the alloy and the specific heat treatment experienced by the alloy were major factors in determining whether these sites have a decisive effect on α-nucleation. In the current study, the Ti‒6Al‒4V‒30Zr alloy had a low dislocation density for the β-field annealing. The TEM and XRD examinations showed that the quenched Ti‒6Al‒4V‒30Zr alloy had homogeneously-distributed nanoscale precipitates of the α″ phase, which will act as the nucleation site for α-phase during aging. Moreover, both α- and α″- phases have two variants, and the angle between the two variants is 90 degrees. As a consequence of the α″-assisted heterogeneous nucleation, a relatively large number of nanosized α precipitates were formed and uniformly distributed within the β-matrix. These nanosized α precipitates create lots of grain boundaries, which inhibit the dislocation motion and result in the strengthening effect.

### 3.4. Structure and Mechanical Properties of the Forged Ti‒6Al‒4V‒30Zr Alloy

**Figure 13:**
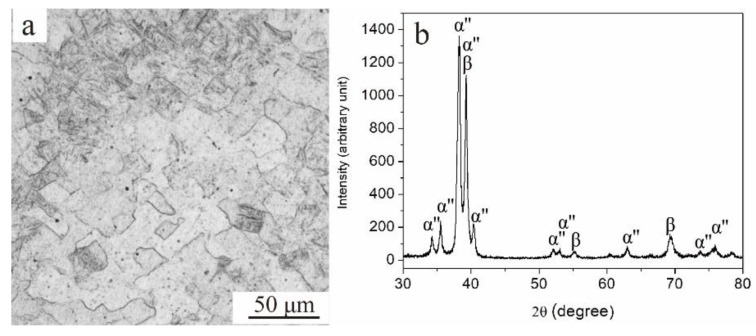
As-forged Ti6Al4V30Zr alloy: (**a**) Optical microscope image; (**b**) XRD pattern.

The overall composition of the as-forged alloy, which was measured via inductively coupled plasma atomic emission spectrometry, is presented in Table 3. The β-transus temperature was 800 °C. The optical microscope image and XRD pattern of the as-forged alloy is shown in Figure 13. As shown in Figure 13, the alloy consisted of β-microstructure and α″-phase, and no spherical α-phase (primary α) can be observed due to the alloy forging in the β-single phase zone.

**Figure 14:**
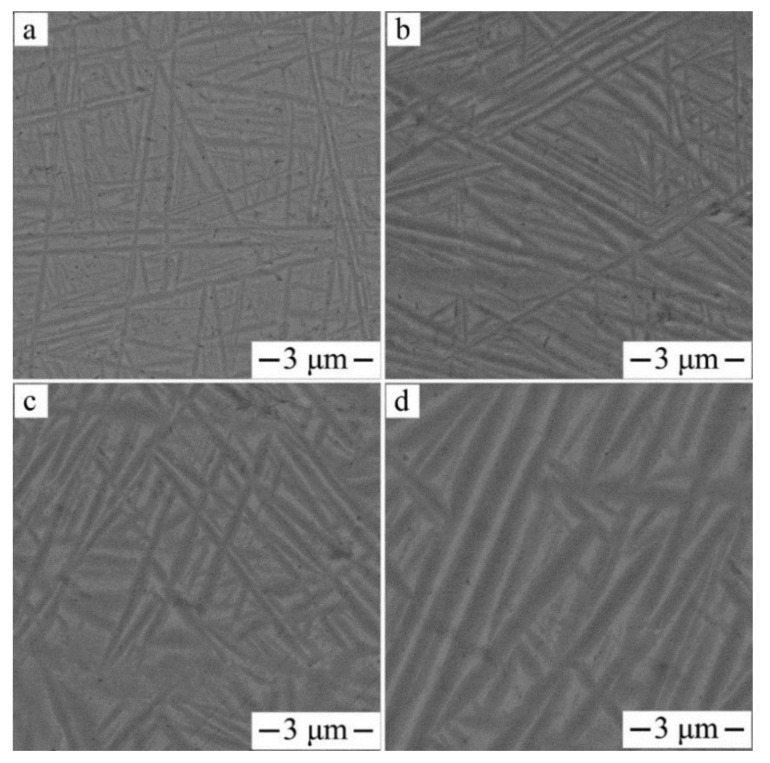
SEM, backscattered electron images of the Ti6Al4V30Zr alloy after solution treated at 820 °C and aged at different temperatures: (**a**) 500 °C; (**b**) 550 °C; (**c**) 600 °C; (**d**) 650 °C.

Figure 14 shows the microstructure of the forged alloy after the β-solution was treated and aged at different temperatures. As shown in Figure 14, each aged alloy displays a typical basket-weave structure of α- and β-phases. When the temperature of the aging treatment was relatively low (500 °C), the needle-like secondary α-phase formed within the β-matrix. Denser dispersion of the α precipitate were present with higher aspect ratios. As the aging temperature increased, the secondary α coarsened, and its volume fraction decreased gradually. Notably, the stagger α precipitates were distributed in the β-matrix with non-uniform size, which is a little different from the α-phase morphology taken from the diffusion multiples. This result may be because forged alloy always introduces numerous grain boundaries, dislocations, and internal stress. These defects increase the strain energy and interfacial energy, which decreases the stability of the system. The assumption is that coarser second α formed at the stage of cooling from the β-field results to finer second α formed at the aging stage.

**Figure 15:**
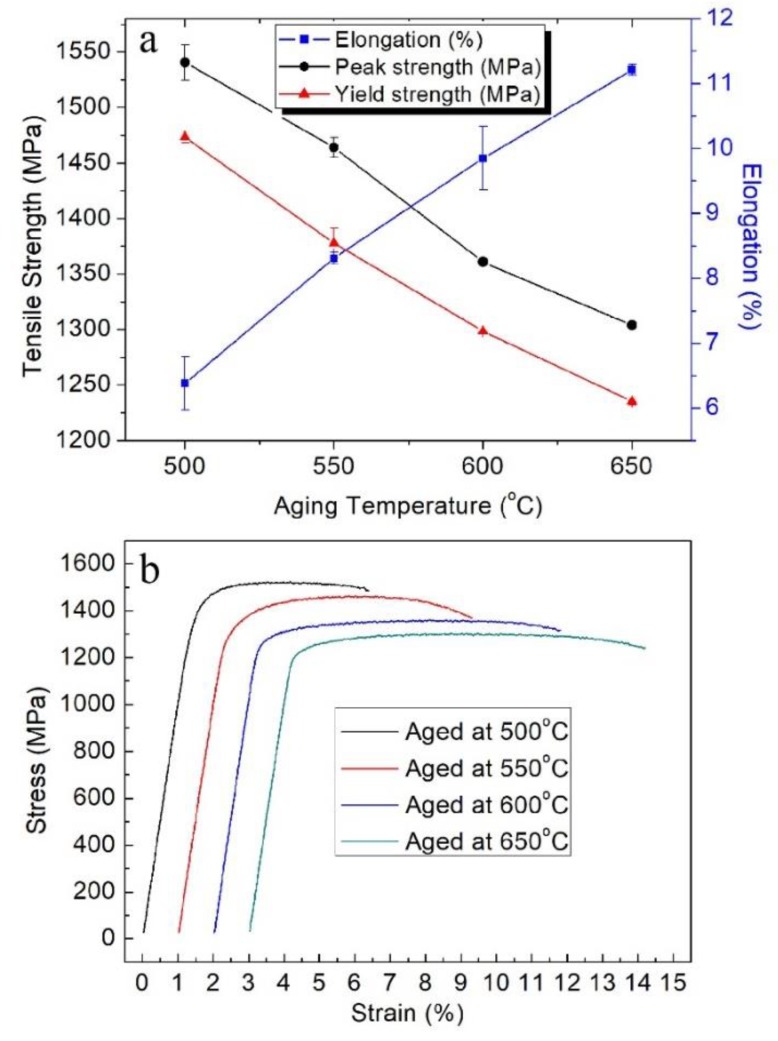
Tensile test results of the alloy solution treated at 820 °C and aged for 6 h: (**a**) tensile properties vary with aging temperature; (**b**) stress-strain curves.

The influence of aging temperature on the tensile properties of Ti‒6Al‒4V‒30Zr alloy after super-transus solution treatment is shown in Figure 15. The tensile properties and stress-strain curves after solution treatment and aging at various temperatures are shown in Figure 15a,b. After aging at 500 °C, the tensile strength and yield strength reached 1540 MPa and 1473 MPa, and the breaking elongation reached 6.4%. When aged at 550 °C, the alloy had a good combination of strength and plasticity, the tensile and strength yield strength were 1464 MPa and 1377 MPa, respectively, with moderate elongation (8.3%). When aged at 650 °C, the alloy had the lowest value of 1235 MPa for yield strength and 1303 MPa for tensile strength, and highest elongation of 11.2%. Overall, increasing the aging temperature decreases tensile and yield strengths of the alloys but increases the total elongation.

The tensile properties of titanium alloy are significantly influenced by the microstructures, especially the size and volume fraction of the secondary α-phase. According to the Hall–Petch formula, the strength of the alloys increase with the decrease in mean width of the α-grains. As shown in Figure 14, when the aging temperature is 500 and 550 °C, the mean thickness of α grains is about 90 and 150 nm, and the strength of the alloys reaches 1540 and 1464 MPa, respectively. Whereas, when the aging temperature increases to 600 and 650 °C, the strength of the alloys decreases to 1360 and 1303 MPa. The increase of grain boundaries density is accompanied by the decrease of grain size. The gain boundaries act as strong obstacles to the dislocation motion and prevent dislocation from moving. The increase in strength resulted from the increase in dislocation density, which is responsible for dislocation tangle.

The breaking elongation percentage of Ti‒6Al‒4V‒30Zr alloy increases with the increase of the α-phase thickness. Major deformation mechanisms in titanium alloys were found to be dislocation glide and tangle [34]. The decrease of the grain size will lessen the thickness of lamellas and lamellar spacing of the α-phase, which will reduce the activity space of dislocation and induce the decrease of breaking elongation [39,40,41]. Moreover, the volume fraction of α-phase is another important factor that has great impacts on mechanical properties. The general trend is that the tensile strength of the alloy increases by increasing the volume fraction of the α-phase, but the elongation decreases. Dehghan-Manshadi also reported that the hardness of Ti-5553 alloy increases monotonously with the increase of volume fraction of α-phase from 0 to 80% [42]. This case might be because the crystal structure of β-phase is bcc, but the structure of α-phase is hcp, and bcc is easier to deform than the hcp structure. Therefore, excessive α-phase will cause plasticity degradation of the alloy. Du et al. [43] reported that, when the volume fraction of α-phase in the alloy is about 60%, the alloy may have the best overall performance.

## 4. Conclusions

In the current study, the diffusion multiples of Ti6Al4V–Ti6Al4V20Fe–Ti6Al4V20Cr–Ti6Al4V20Mo–Ti6Al4V45Zr were manufactured to obtain a large composition space. Scanning electron microscopy, EPMA, and a microhardness system were combined to determine the relationships among the composition, microstructure, and hardness of these alloys. The microstructure, mechanical properties, and phase transition of Ti‒6Al‒4V‒30Zr alloy were also investigated. The following findings are reported:

(1) When the Zr content increased to 30 wt.%, the quenched α′ phase decreased and disappeared, and the lamellar thickness of α-phase decreased to nanoscale, which resulted to maximum hardness of the quenched and aged alloy. When the Zr content further increased, the lamellar thickness of the α-phase increased, and the hardness decreased.
(2) Both HAADF-STEM and XRD were used to determine the microstructure and phase composition after quenching and aging of the Ti‒6Al‒4V–30Zr alloy. The quenched alloy displayed homogeneously distributed nanoscale α″ phases, which will be the nucleation site of α-phase during aging.
(3) The forged Ti–6Al–4V–30Zr alloy in the β-solution and aging showed the non-uniform secondary α-phase distribution in the matrix. By increasing the aging temperature from 500 °C to 650 °C, the tensile strength and yield strength of the alloys rose, but the total elongation decreased. The lamella thickness and volume fraction of the α-phase were the major factors that had great impacts on the mechanical properties. As the lamella thickness and volume fraction of the α-phase increased, the tensile strength of the alloy decreased, but the elongation decreased.

## Figures and Tables

**Table 1:** Chemical composition of the as-received Ti–6Al–4V and Ti–6Al–4V–45Zr (wt.%).

Element	Al	V	Zr	Fe	O	N	C	Ti
Ti6Al4V	6.37	3.61	-	0.13	0.18	0.02	0.02	Bal.
Ti6Al4V45Zr	6.47	3.63	44.85	0.15	0.11	0.02	0.01	Bal.

**Table 2:** Chemical composition of the α phase and the β phase in the Ti6Al4V30Zr alloy after aging at 600/650 °C (as shown in Figure 11d and Figure 12d).

Aging Temperature	Position	Phase	wt.% V	wt.% Zr	wt.% Ti	wt.% Al
600 °C	1 #	α	1.9	34.4	56.7	7
	2 #	α	1.5	35.6	55.7	7.1
	3 #	β	13	28.8	55.5	2.7
	4 #	β	14.5	28.8	54.3	2.4
650 °C	1 #	α	1.8	34.9	55.9	7.3
	2 #	α	1.9	34.5	56.2	7.4
	3 #	β	10.5	34.1	52.6	2.7
	4 #	β	10.3	33.2	52.5	2.8

**Table 3:** The chemical composition of titanium ingot (balance Ti, wt.%).

Element	Al	V	Zr	O	N	C	Ti
Ti6Al4V30Zr	5.9	3.7	29.3	0.16	0.012	0.01	Bal.
